# Major bioactive chemical compounds in Astragali Radix samples from different vendors vary greatly

**DOI:** 10.1371/journal.pone.0254273

**Published:** 2021-07-07

**Authors:** Bijay Kafle, Jan P. A. Baak, Cato Brede

**Affiliations:** 1 Department of Chemistry, Bioscience and Environmental Engineering, University of Stavanger, Stavanger, Norway; 2 Department of Pathology, Stavanger University Hospital, Stavanger, Norway; 3 Dr Med Jan Baak AS, Tananger, Norway; 4 Department of Medical Biochemistry, Stavanger University Hospital, Stavanger, Norway; Tallinn University of Technology, ESTONIA

## Abstract

The worldwide traditional Chinese medicine (TCM) herbs sales figures have increased considerably to 50 billion US$ (2018). Astragali Radix (AR) is amongst the most often sold TCM herbs; sales in the European Union (EU) need European Medicines Agency (EMA) approval. However, comparisons of characteristic bioactive molecules concentrations in AR from different EU vendors are lacking. This study uses liquid chromatography-tandem mass spectrometry (LC-MS/MS) with standard addition to evaluate the influence of different sample and preparation types and ammonia treatment on bioactive molecules concentrations in AR. We also compare AR samples from different EU-vendors. Astragaloside IV (AG-IV), ononin and calycosin 7-O-β-D-glucoside concentrations were higher in root powder samples when extracted with boiled water than with ultrasonication using 70% methanol. AG-IV content was by far the highest in granulates from vendor 1 (202 ± 35 μg/g) but very low in hydrophilic concentrates from vendor 1 (32 ± 7 μg/g) and granulates from vendor 4 (36 ± 3 μg/g). Ammonia-treatment significantly increased AG-IV concentrations in all samples (e.g., to 536 ± 178 μg/g in vendor 1 granulates). Comparable effects were found for most other bioactive molecules. AG-IV and other bioactive molecules concentrations differed strongly depending on sample types, extraction processes, ammonia treatment-or-not and especially between different vendors samples. Ammonia-treatment is debatable, as it is supposed to convert other astragalosides, to AG-IV. The results indicate that routine quantitative analysis of major bioactive compounds present in AR, helps in quality control of AR and to guarantee the quality of commercial products.

## Introduction

The annual worldwide sales figures for traditional Chinese medicine (TCM) herbs have tremendously increased over the past decade to about 50 billion US$ in 2018 [1] and is expected to further increase significantly up to 115 billion in 2025 [2]. TCM herbs are used to maintain and strengthen health, prevent and treat diseases and also to prevent serious side effects caused by western medicines such as chemotherapy [3].

Astragali radix (AR), the dried roots of *Astragalus mongholicus* Bunge and *Astragalus propinquus* Schischkin, family Leguminosae, (known in China as Huang Qi) is one of the strongest and most widely used herbs of the subgroup “tonifying TCM herbs”. AR is regarded by many TCM specialists as one of the most important and often used herbs. Indeed, there is a broad therapeutic spectrum for AR, such as improving survival, strongly reducing the side effects of chemotherapy and improving the quality of life (QOL) of metastatic non-small cell lung cancer patient [4, 5] and colorectal cancers of any stage [6, 7]. Other experimental studies claimed that AR possesses many different biological qualities, such as anticancer, anti-virus, anti-asthma, protection from radiation [8], antioxidant capacity, immunomodulatory effects [9], anti-oxidative, cardiovascular and liver protection [10].

The potential bioactivity of AR is due to the presence of bioactive compounds such as isoflavonoids, saponins, polysaccharides, amino acids and trace elements [8] of which astragalosides (AG) (I-VIII) are considered characteristic bioactive therapeutic compounds. Astragaloside saponins showed anti-aging, anti-inflammatory and anti-tumor effects [11], can act as an anti-cancer agent by targeting the non-steroidal anti-inflammatory drugs-activated gene (NAG-1) during its regulation of apoptotic activities [12] and inhibit cell proliferation in human colon cancer cell lines and tumor xenografts [13]. Astragaloside IV (AG-IV) showed potent anti-hepatitis B virus activity [14] and inhibited the growth, invasion and migration of lung cancer cell [15]. Experiments on cycloastragenol (an aglycone of AG-IV), revealed that it can delay the cellular aging process by increasing telomerase activity [16]. Treatment of neurodegenerative diseases can be facilitated by use of cycloastragenol because of its telomerase activating and cell proliferating properties [17]. Cycloastragenol can be produced metabolically by intestinal bacteria or by acid hydrolysis of AG-IV [17, 18] and even by metabolic conversion of other astragalosides (AG-I, AG-II and AG-III) [19]. However, studies of AR as a single herb are nearly exclusively performed in cell cultures and animal models [20–22].

To the best of our knowledge, there have only been a few studies on the pharmacological effects of AR in humans, including renal protection effects [23] and against sudden hearing loss [24]. Formononetin and AG-IV have been tested in healthy human volunteers and appeared totally safe. Pharmacokinetic analysis of formononetin in healthy Chinese volunteers (n = 9), showed the highest plasma concentration of 2.39 ± 1.20 ng/mL 2.15 hours after ingestion of 30 g ultrafine granular powder of AR [25]. Moreover, intravenous infusion of astragaloside IV was well tolerated in a single and in multiple administration(s) of 54 mg: without accumulation of astragaloside IV in plasma. Urine excretion was not the major route of elimination [26], probably metabolically transformed.

Despite lacking scientific evidence for effects in humans, AR has been used in China for hundreds of years. The pharmacopeial recommendations are therefore based on extensive empirical experience. The daily therapeutic dosage of AR advised in humans varies from 9–30 g [27]. Side effects are said to be minimal, if any. It is also allowed in pregnancy and to our knowledge no publications of side effects in pregnancy are known. Yet, these empirical and clinical practices are not in agreement with the aim and statement, that the selection of TCM herbs by TCM practitioners should be in line with scientific standards and management systems.

Assays for quality control of AR are described in Chinese, European & Taiwanese pharmacopoeias. Previously, the amount of AG-IV after assay present in AR, should not be less than 0.04% (w/w) to pass the quality test [28]. However, he recent edition of Chinese pharmacopoeia 2020 stated, that the threshold of AG-IV must not be less than 0.08% (w/w). The pharmacopoeias also advise that the pharmacopeial assays should use ammonia during extraction. However, ammonia is supposed to convert many astragalosides (without proven biological effect) into AG-IV [29]. With oral intake by humans, such a conversion may not occur. To reach the 0.08% acceptance limit for AG-IV, will therefore also depend on the concentration of other non-AG-IV astragalosides, but does not say anything about it´s true AG-IV biological activity.

In Europe, the 2004/24/EC directive includes herbal medicinal products. TCM products sold in the European Union (EU) [30], need approval by the European Medicines Agency (EMA) and adherence to their guidelines [31]. Despite the abovementioned shortfall in scientific evidence, AR is allowed by the EMA and sold in the EU.

Inevitably, the phytochemical composition of the herbs grown naturally varies due to differences between plant origin, geographical conditions and growth environment [9, 32]. There are also differences between different Astragalus species [33]. Post-harvesting factors such as processing and preparation of the herbs, may further influence the final composition of bioactive components [10, 34]. It therefore could well be, that AR sold by different vendors in the EU for medical purposes, vary in quality and composition. There are many products of AR available in the market of EU in which the composition of bioactive compounds is not stated. AR prescriptions used by different EU health care providers may therefore vary, be suboptimal and lead to suboptimal therapeutic effects. Quantification of bioactive molecules in AR samples is essential. It is also of great importance to know the effects of the factors influencing the composition of the final AR product, to know the real amounts of phytochemicals ingested in such experiments.

In the present study, we investigated whether there is any variation in the concentrations of major isoflavones and AG-IV in different AR herbal types (raw herbs, granulates, tablets, and hydrophilic concentrates) sold in the EU. These commercial samples are widely used in the European Union and are indeed representative for TCM applications. Furthermore, we assessed the concentrations of phytochemicals in dry root samples when using two different extraction methods (conventional boiling in water and a much faster technique sonication using 70% methanol). We also investigated how the measured concentration levels were affected by adding ammonia solution to the extracts. Finally, we analysed the quantity of these bioactive molecules in AR sold by different EU vendors.

Different methods have been used to determine major bioactive isoflavonoids and saponins present in AR samples using ultraviolet (UV) and tandem mass spectrometry (MS/MS) detection after liquid chromatographic separation [35, 36]. Quantification in the present study was performed with a validated LC-MS/MS method as previously described [36]. The method utilized standard addition quantification to overcome the issue of lack of isotopic labelled internal standards. This method greatly improves the measurement accuracy of phytochemicals in AR herbs.

## Materials and methods

### Chemicals and reagents

**Fig 1:**
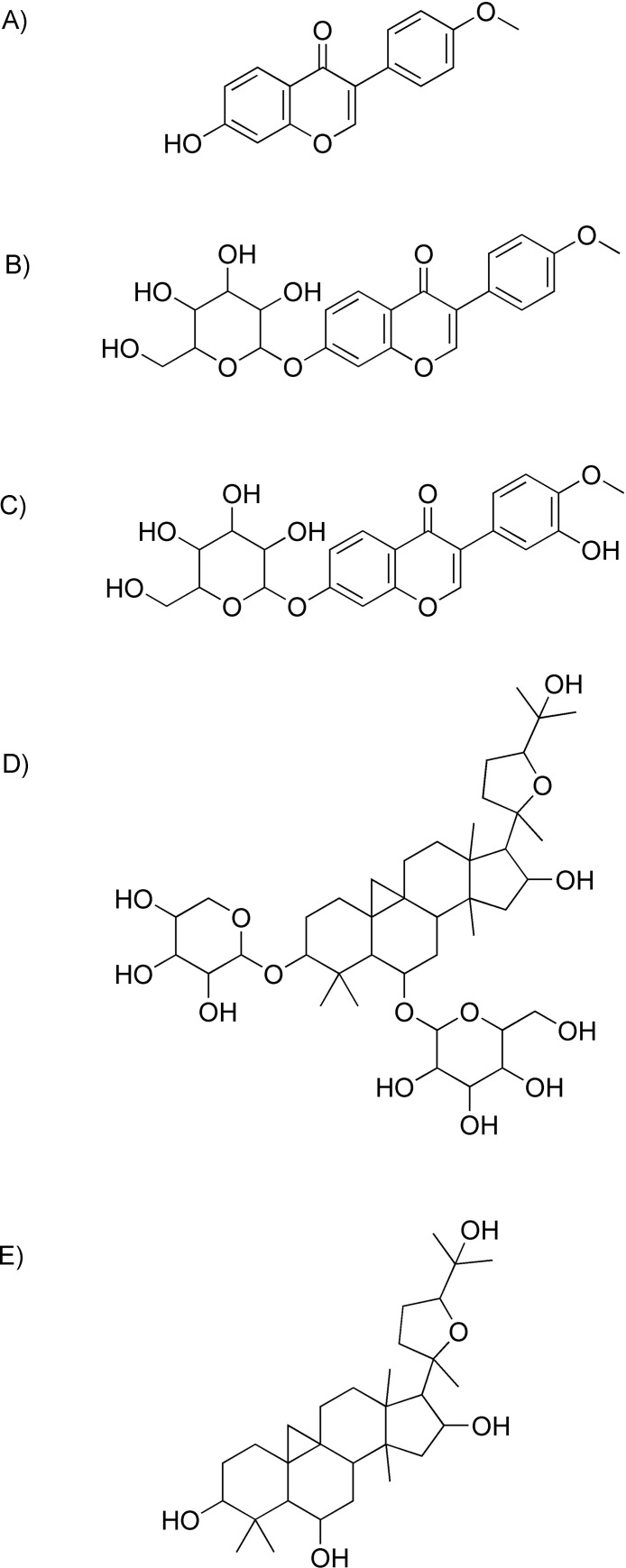
Molecular structures of A) formononetin (C_16_H_12_O_4_), B) ononin (C_22_H_22_O_9_), C) calycosine 7-O-β-D-glucoside (C_22_H_22_O_10_), D) astragaloside IV (C_41_H_68_O_14_) and E) cycloastragenol (C_30_H_50_O_5_).

The reference standard chemical compounds of Formononetin (≥ 98%, lot no: BCBZ9069) and Cycloastragenol (≥ 98%, lot no: SLBM2014V) were purchased from Sigma-Aldrich Co. (St. Louis, USA). Astragaloside IV (98%, lot no: PRF90922502), Ononin (98%, lot no: PRF9060501) and Calycosin 7-O-β-D-glucoside (98%, lot no: PRF8071905) were purchased from Chengdu Biopurify Phytochemicals Limited (Sichuan, China). Molecular structures of these major bioactive compounds are provided (Fig 1). Methanol, acetonitrile and formic acid were of LC-MS grade obtained from VWR International AS (Oslo, Norway). All other reagents were of analytical grades. Purified water was prepared using Elga-purelab Flex water purification system (High Wycombe, UK).

### Sample extracts

The AR samples were purchased from four different vendors as dried roots, granulates, hydrophilic concentrate, capsules or tablets (Table 1). AR granulates (Kaiser Pharmaceutical Taiwan), Dried roots (Pharmaceutical wholesalers) and Hydrophilic concentrate (Conforma NV, Belgium) were obtained from the Natuurapotheek (NA) (vendor 1), Pijnacker, the Netherlands. Similarly, AR capsules (Swanson Health Products, USA) were obtained from Authentic Produce Limited (AP) (vendor 2), Jersey, UK, AR tablets were obtained from Seven Forest (SF) (vendor 3), Seattle, USA, and AR granulates (Green Nature, Hong Kong, China) were obtained from Chinese Medical Centre (CMC) (vendor 4), Amsterdam, the Netherlands.

**Table 1:** Sample extracts.

Sample ID	Sample/Vendors*	Lot no.	Weight	Extraction conditions	Final volume (mL)
A	Granulates/NA	GR– 77/3/19	5 g	Boiled in water	20
A1	Granulates/NA	GR– 77/3/19	5 g	Ultrasonication	20
B	Dried root powder/NA	HB– 01350146	5 g	Ultrasonication	20
B1	Dried root powder/NA	HB– 01350146	2.5 g	Boiled in water	40
C	Hydrophilic concentrate/NA	HC-17J10/V90291	5 g	No extractions	6.009
			~ 6.009 mL		
D	Dried root capsules/AP	C236378	5 g	Ultrasonication	20
E	Tablets/SF **	G8855	5 g	Ultrasonication	20
F	Granulates/CMC	20–211231	5 g	Ultrasonication	20

**12% Astragalus Root w/w per tablet.

* NA (Natuurapotheek), AP (Authentic Produce), SF (Seven Forest), CMC (Chinese Medical Centre).

The dried roots and tablets were crushed to powder using a mortar and pestle. Dry root powder was acquired from decapsulated capsules. Powdered samples were extracted either by boiling the samples in water for 60 min or by ultrasonication using 70% methanol at 40°C for 60 min using Branson Ultrasonicator (Danbury, USA). To remove impurities, all sample extracts were centrifuged at 4000 rpm for 10 minutes twice using an Eppendorf Centrifuge 5702 (Hamburg, Germany). The sample extracts were dried using an IKA HB 10 evaporator (Ohio, USA). The dried extracts were reconstituted to final volume of 20 mL with pure methanol. The liquid samples of hydrophilic concentrations were analysed without extraction after centrifugation, using a liquid density of 0.83 g/mL.

### Ammonia treatment of sample extracts

To study the effects of ammonia on bioactive chemical constituents of AR, specially AG-IV, sample extracts were analysed after treatment by addition of an equal volume of 20% ammonia solution. All samples were centrifuged at 4000 rpm for 10 minutes, before injection into LC- MS/MS.

### Standard solutions

The reference standards of all five chemical compounds were weighed and dissolved in methanol to make a solution with final concentration of 1 mg/mL. Ononin and formononetin were dissolved by adding 4–5% acetone in methanol and heating gently to 40°C. The stock solutions of each standard compound were prepared with a final concentration of 0.1 mg/mL in methanol. The standard dilutions for all compounds were: 0.3125, 0.625, 1.25, 2.5, 5, 10 and 20 μg/mL. For standard addition, diluted samples were spiked to compound concentrations of 0, 0.5, 1 and 2 μg/mL.

### LC-MS/MS analysis

The instrumental analysis was performed by a validated method as previously described [36]. Briefly, the LC-MS/MS instrument was an Acquity UPLC coupled with a Quattro Premier XE triple quadrupole mass spectrometer (Waters Corporation, Massachusetts, USA). Phytochemicals were separated on a reverse phase BEH C18 column (100 mm long x 2.1 mm ID) with 1.7μm particle size and 130 Å pore size (Waters) by using a mobile phase gradient of 0.2% formic acid mixed with methanol. Positive electrospray ionization (ESI+) and MRM were applied for detection. Samples were diluted ten times before analysis, and phytochemicals were accurately quantified by standard addition.

## Results

The influence of the extraction process and different sample types were studied first. The AG-IV concentration in dried roots from vendor 1 when boiled in water was significantly higher (almost double) than in methanolic extracts using ultrasonication (63 ± 6 μg/g vs. 32 ± 7 μg/g). However, the concentration of formononetin was lower in boiled water extract (89 ± 6 μg/g) than in 70% methanol extracts using ultrasonication (133 ± 38 μg/g), although the high standard deviation in the latter results show that the effect varied greatly. The concentration of two other isoflavonoids was higher in boiled water extracts than in extracts prepared by ultrasonication (ononin: 126 ± 3 μg/g vs. 49 ± 6 μg/g; calycosin 7-O-β-D-glucoside 384 ± 24 μg/g vs. 118 ± 23 μg/g). This shows the strong influence of the extraction process and solvents used on the concentration estimates of the bioactive compounds. Boiled water extractions, the classical extraction manner showed superiority over (the faster) 70% methanol extraction using ultrasonication, when AG-IV, ononin and calycosin are the target molecules.

As expected, the granulates sample from vendor 1 had much (>3x) higher concentrations of AG-IV than the raw root samples from the same company. Interestingly, almost equal amounts of AG-IV were measured after granulates samples were extracted using boiled water, as with ultrasonication extraction in 70% methanol (200 ± 70 μg/g vs. 202 ± 35 μg/g).

Ammonia treatment resulted in a manifold increase in the concentration of AG-IV in all samples. In fact, ammonia treatment was necessary to approach the required minimum concentration limit (0.08% w/w = 800 μg/g) for AG-IV specified in the Chinese pharmacopoeia (Table 2), and even then, the threshold level was not reached in any of the samples.

**Table 2:** Pharmacopeial limit of compounds to be present in AR.

Pharmacopoeia	Compounds	Required minimum concentration limit (% w/w)	Detectors
Taiwan Herbal Pharmacopoeia (2^nd^ Ed. 2016)	Astragaloside IV	0.04	ELSD
Chinese Pharmacopoeia (2020)	Astragaloside IV	0.08	ELSD
Chinese Pharmacopoeia (2010)	Calycosin 7-O-β-D-glucoside	0.02	ELSD
European Pharmacopoeia (7.0, 2011)	Astragaloside IV	0.04	ELSD
Japanese Pharmacopoeia (17^th^ Ed. 2016)	Astragaloside IV	Not specified	TLC

* All the samples tested with ELSD used ammonia for extraction.

In granulates samples from vendor 1, the concentration of AG-IV increased from 202 ± 35 μg/g to 536 ± 178 μg/g after ammonia solution treatment. Similarly, in dried roots from vendor 1, the concentration of AG-IV was much higher after ammonia treatment (32 ± 7 μg/g versus 315 ± 137 μg/g). With ammonia treatment, the concentrations of AG-IV were 369 ± 95 μg/g and 306 ± 71 μg/g in dried root samples from vendor 2 and granulates from vendor 4 respectively. In contrast, isoflavonoids concentration did not show a clear trend but fluctuated after treatment with ammonia (i.e., increased in some but decreased in other samples), Table 3.

**Table 3:** Concentrations (μg/g) in different samples of isoflavones and astragaloside IV determined by LC-MS/MS using standard addition.

Method	LC-MS/MS, Standard addition	
Sample	10 x diluted sample extract	10 x 2 diluted sample extract treated with 20% ammonia
**Sample A (NA Granulates, boiled water extractions)**		
Astragaloside IV	200 ± 70	NA
Formononetin	22 ± 2	NA
Ononin	41 ± 2	NA
Calycosin 7-0-β-D-glucoside	241 ± 53	NA
**Sample A1 (NA Granulates, 70% methanol extraction using ultrasonication)**		
Astragaloside IV	202 ± 35	536 ± 178
Formononetin	29 ± 4	25 ± 5
Ononin	35 ± 3	13 ± 8
Calycosin 7-0-β-D-glucoside	118 ± 20	121 ± 24
**Sample B (NA Dried root powder, 70% methanol extraction using ultrasonication)**		
Astragaloside IV	32 ± 7	315 ± 137
Formononetin	133 ± 38	78 ± 14
Ononin	49 ± 6	36 ± 8
Calycosin 7-0-β-D-glucoside	118 ± 23	216 ± 80
**Sample B1 (NA Dried root powder, boiled water extractions)**		
Astragaloside IV	63 ± 6	NA
Formononetin	89 ± 6	NA
Ononin	126 ± 3	NA
Calycosin 7-0-β-D-glucoside	384 ± 24	NA
**Sample C (NA Hydrophilic concentrate, no extractions)**		
Astragaloside IV	32 ± 7	104 ± 28
Formononetin	29 ± 1	21 ± 8
Ononin	36 ± 3	7 ± 0
Calycosin 7-0-β-D-glucoside	121 ± 23	47 ± 11
**Sample D (AP Capsules, 70% methanol extraction using ultrasonication)**		
Astragaloside IV	78 ± 11	369 ± 95
Formononetin	47 ± 2	57 ± 8
Ononin	109 ± 30	112 ± 0
Calycosin 7-0-β-D-glucoside	336 ± 104	13 ± 2
**Sample E (SF Tablets, 70% methanol extraction using ultrasonication)**		
Astragaloside IV	9 ± 2	221 ± 67
Formononetin	5 ± 0	6 ± 0
Ononin	8 ± 1	31 ± 23
Calycosin 7-0-β-D-glucoside	17 ± 2	14 ± 1
**Sample F (CMC Granulates, 70% methanol extraction using ultrasonication)**		
Astragaloside IV	36 ± 3	306 ± 71
Formononetin	54 ± 1	84 ± 2
Ononin	25 ± 3	18 ± 4
Calycosin 7-0-β-D-glucoside	120 ± 4	92 ± 11

NA = Not Analysed, Sample E (Tablets from Seven Forest contained 12% of astragalus root per 1 g tablet).

We also compared samples from different vendors without ammonia treatment, to avoid measuring artificially high levels of AG-IV with no medical significance, and rather obtain levels that would represent the eventual therapeutic effect of the samples with oral intake in humans.

There were huge and significant differences in the concentrations of bioactive molecules in samples from the different vendors 1, 2, 3 and 4. The most important bioactive compound (AG IV) was highest in granulates samples from vendor 1 (202 ± 35 μg/g). The second highest content was nearly 60% lower (in dried roots in capsules, from vendor 2, 78 ± 11 μg/g). Hydrophilic concentrates from vendor 1 and granulates from vendor 4 had very low concentrations. AG IV concentration was only 9 ± 2 μg/g in tablet samples from vendor 3, which was the lowest from all samples. However, this was not surprising, as the vendor indicated in the specifications that the content of AR was very low.

Calycosin 7-O-β-D-glucoside (384 ± 24 μg/g) and ononin (126 ± 3 μg/g) are highest in boiled root extracts from vendor 1 followed by capsules from vendor 2 when extracted by solvent (70% methanol), while formononetin (133 ± 38 μg/g) is highest in roots from vendor 1 when extracted by solvent (70% methanol). The concentration of isoflavonoids in boiled water extracts of granulates from vendor 1 are: formononetin 22 ± 2 μg/g, ononin 41 ± 2 μg/g and calycosin 7-O-β-D-glucoside 241 ± 53 μg/g. The isoflavonoids contents in hydrophilic concentrate from vendor 1 and granulates from vendor 4 were much less than half the concentrations of isoflavonoids present in raw roots from vendor 1. The concentration of all measured compounds in tablets from vendor 3 were very low, but again, this was as expected, because these tablets contain only 12% of astragalus roots present per g tablet material. The obtained concentration of compounds was calculated per total amount of astragalus root present in the tablets, to measure whether they meet the pharmacopeial threshold standards. Cycloastragenol was not detected in any of the samples tested, in agreement with the fact that it is naturally absent in AR [18].

**Fig 2:**
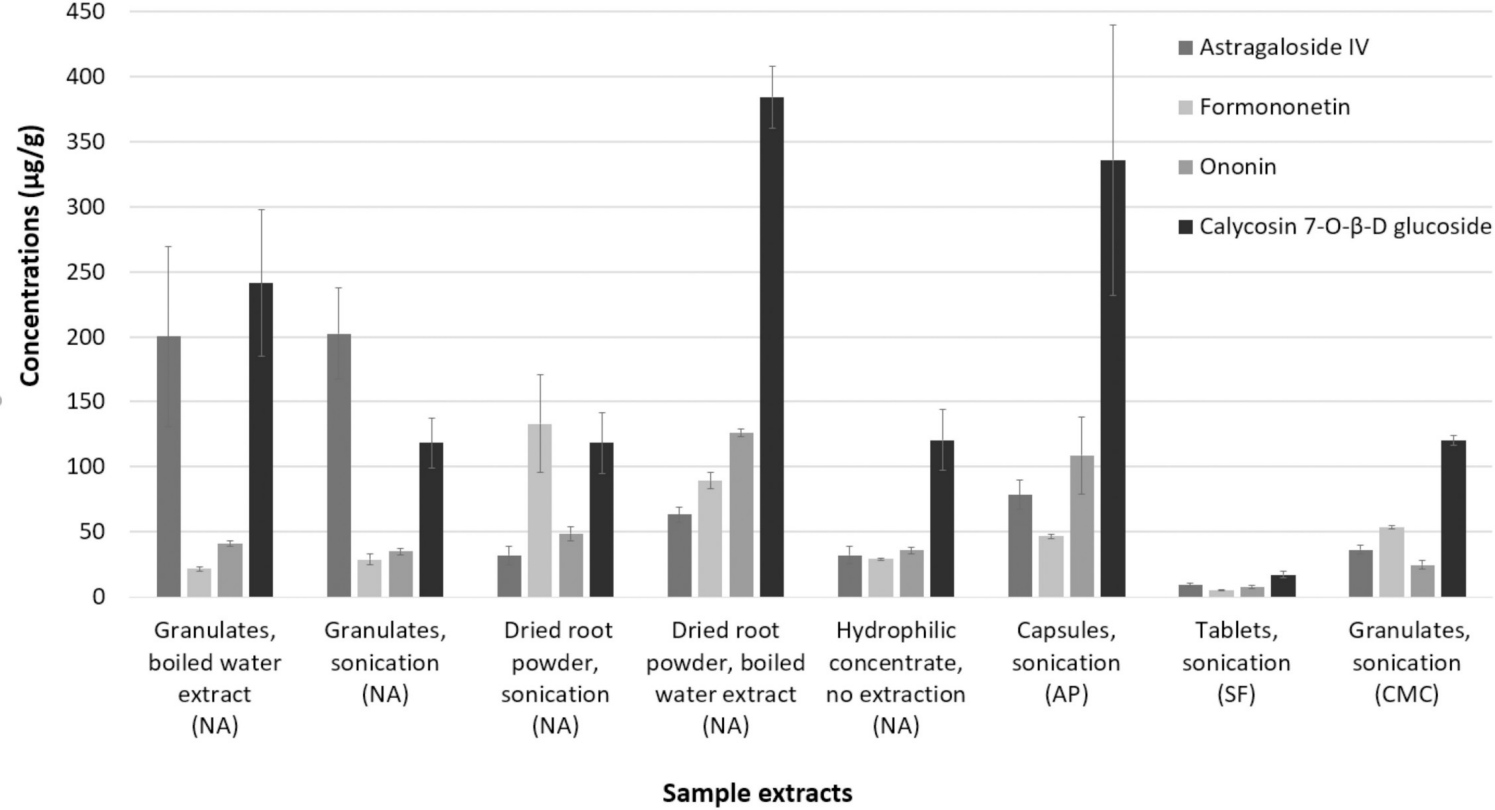
Histogram showing the concentrations of biologically active components (mean ± SD) present in AR samples from different vendors.

Surprisingly, the granulate samples from vendors 1 and vendor 4 have hugely different concentration of AG-IV (almost 5.5-fold more in vendor 1 granulate). The isoflavonoids concentrations were similar. There were also variations in chemical composition in dried raw roots from vendor 1 and root powder in capsules obtained from vendor 2, i.e., variation in the same type of herbal samples from different vendors. The differences in the concentrations of the bioactive molecules in the samples from different vendors, are summarized in Fig 2.

## Discussion

Over the past decade, there is a strong increase in interest in the use of TCM herbal medicines worldwide, including the EU. TCM herbs can be bought on the internet and then are often inexpensive, whereas EU-approved herbs can be (much) more costly. It can be difficult for medical prescribers to identify high quality commercially available TCM herbal products, when the characteristic bioactive molecules are not known [33], as is the case with all the samples studied, sold by the EU vendors.

Pharmacopoeias describe procedures and analytical methods for quality control (QC). The Chinese pharmacopoeia (2010) specifies only two species of Astragalus as *A*. *membranaceous* (Fisch.) Bge. or *A*. *membranaceous* var. *mongholicus* (Bge.) Haiso for therapeutical usage [34]. The quality of these herbs may vary with respect to environmental conditions, geography, age of plant and different species [33, 34]. The recent 2020 edition of the Chinese pharmacopoeia indicates that AG-IV content should not be less than 0.08% to pass the quality limit. Interestingly, none of the samples studied fulfilled this condition, not even when analysed after using ammonia during sample preparation. These results indicate certain shortcomings of the chromatographic methods prescribed by the pharmacopeias for AR samples [37]. Selection of more reliable detector type is very important for accurate quantification. Studies have used the non-selective ELSD detector which depends upon the retention time of compounds, and since different molecules can co-elute together, it is possible that the identification and quantification of target compounds may be compromised [38]. To avoid these problems, we previously developed a very accurate, sensitive, and reproducible LC/MS-MS method with standard addition quantification [36], which was also used in the present study.

Another debatable point is ammonia treatment of the sample extracts (as recommended in different Pharmacopoeias). Indeed, our results showed that such a process to analyse AR helps to approach the lowest concentration threshold of 0.08% (w/w). One study reported on the analysis of AR samples without ammonia treatment using the standard ELSD detector showing in average of 0.016% (0.08 mg of AG-IV in 0.5 g of AR samples) of AG-IV [39]. These values are similar to the results obtained in our present study without ammonia treatment. The sharp increase in AG-IV concentration by adding ammonia contrasts the much lower concentration of the naturally available characteristic bioactive AG-IV. The increased AG-IV concentration after ammonia treatment therefore is probably due to hydrolytic conversion of other non-AG-IV astragalosides without known medical function [29, 40]. A similar hydrolysis may not occur in the human digestive tract when AR is taken orally. Hence, until we know more about metabolism of different astragalosides in humans, the therapeutic intake levels of AG-IV should be calculated by using ammonia-free extraction measurement results.

Surprisingly, the content of bioactive molecules in AR samples measured by LC/MS-MS using standard addition, varied strongly in samples from different vendors, even when the samples consisted of granulates [9]. In the present work, we applied LC-MS/MS with multiple reaction monitoring (MRM) [22, 41] to ensure a high degree of selectivity and sensitivity in the analysis. Samples were diluted, and the phytochemicals quantified by standard addition, which compensated for ion suppression matrix effects [42, 43] and provided accurate results in the absence of isotopic labelled internal standards [36]. Standard addition therefore provides a viable solution when isotopic labelled internal standards are not available, as is often the case when measuring phytochemicals by LC-MS/MS. There was no cycloastragenol present in any of the commercial samples studied, might be in a lower concentration than the detection limit or naturally absent [18]. Cycloastragenol, which is the aglycon of AG-IV, may be possibly metabolized from AG-IV and other astragalosides, in the human digestive tract [19].

The dried concentrated extracts (granulates) from Vendor 1, are probably easily digested in the human digestive tract, because similar concentrations of AG-IV were found when dissolving granulates in boiling water as with solvent extraction. However, more quantity of bioactive compounds such as AG-IV, ononin and calycosin 7-O-β-D-glucoside were extracted from dried roots using boiled water. This is reasonable because granulates are pre-processed samples. More extensive studies are required to generalize the superiority of traditional boiled water extraction method to other chemical extraction methods. Since methanol and ammonia cannot be used during sample preparation for oral intake, the concentration thus obtained cannot be correlated to the concentration available for systemic absorptions. Granulate samples from vendor 1 have the highest concentration of the most important bioactive molecules. Granulate samples from vendor 4 were cheaper but had 6 times lower bioactive molecules concentrations.

QC studies of TCM herbs will provide baseline data for pharmacokinetics and dose-response relationship during therapeutic use. Simple, preferably non-invasive tests such as analysis of human saliva after administration of TCM samples can be evaluated as a marker for blood concentrations. If these results are positive, saliva testing would make personalized dosaging of TCM herbs in individual patients much easier.

In conclusion, the concentrations of essential bioactive molecules of AR vary greatly between EU vendors. Indeed, the use of ammonia during sample preparation significantly increased the concentrations of AG-IV. Before we know more about hydrolytic conversion of astragalosides in humans, the therapeutic intake levels cannot be estimated from artificially increased testing levels of AG-IV produced by ammonia treatment. Our findings will be relevant for future TCM therapy and for medical research with these herbs.

## Supporting information

S1 File (XLSX)

## References

[1] Pinghui Z. Traditional Chinese medicine closes in on US$50 billion market with long-awaited nod from WHO. South China Morning Post. China: South China Morning Post. [Cited 2020 November 11] Available from: https://www.scmp.com/news/china/society/article/2166278/traditional-chinesemedicine-closes-us50-billion-market-long

[2] Xu J, Xia Z. Traditional Chinese medicine (TCM)–Does its contemporary business booming and globalization really reconfirms its medical efficacy & safety?. Medicine in Drug Discovery. 2019; 1: 1–5. e100003. doi: 10.1016/j.medidd.2019.100003

[3] Guo H, Liu JX, Li H, Baak JPA. In metastatic non-small cell lung cancer platinum-based treated patients, herbal treatment improves the quality of life. A prospective randomized controlled clinical trial. Frontiers in Pharmacology. 2017; 8: 1–11. e454 doi: 10.3389/fphar.2017.00001 28769793 PMC5511837

[4] Baak J, McCulloch M, Hempen C-H. TCM in metastatic lung cancer: prognosis and complementary treatment of advanced pulmonary non-small-cell lung cancer (NSCLC). The European Journal of Oriental Medicine. 2015; 8(2): 8–24.

[5] McCulloch M, See C, Shu X-J, Broffman M, Kramer A, Fan W-Y, et al. Astragalus-based Chinese herbs and platinum-based chemotherapy for advanced non-small-cell lung cancer: meta-analysis of randomized trials. Journal of Clinical Oncology. 2006; 24(3): 419–30. doi: 10.1200/JCO.2005.03.6392 16421421

[6] Tan KY, Liu CB, Chen AH, Ding YJ, Jin HY, Seow-Choen F. The role of traditional Chinese medicine in colorectal cancer treatment. Techniques in Coloproctology. 2008; 12(1): 1–6. doi: 10.1007/s10151-008-0392-z 18512006

[7] McCulloch M, Broffman M, Laan M, Hubbard A, Kushi L, Abrams DI, et al. Colon cancer survival with herbal medicine and vitamins combined with standard therapy in a whole-systems approach: ten-year follow-up data analyzed with marginal structural models and propensity score methods. Integrative Cancer Therapies. 2011; 10(3): 240–59. doi: 10.1177/1534735411406539 21964510 PMC4081504

[8] Chen Z, Liu L, Gao C, Chen W, Vong CT, Yao P, et al. Astragali Radix (Huangqi): A promising edible immunomodulatory herbal medicine. Journal of Ethnopharmacology. 2020; 258: 1–18. e112895. doi: 10.1016/j.jep.2020.112895 32330511

[9] Chen H, Zhou X, Zhao Y, Gong XJ, He Y, Ma F-W, et al. HPLC-DAD-ELSD combined pharmacodynamics and serum medicinal chemistry for quality assessment of Huangqi granule. PloS One. 2015; 10(4): 1–15. e0123176. doi: 10.1371/journal.pone.0123176 25915040 PMC4411121

[10] Gong AGW, Duan R, Wang HY, Kong XP, Dong TTX, Tsim KWK, et al. Evaluation of the pharmaceutical properties and value of Astragali Radix. Medicines. 2018; 5(2): 1–16. doi: 10.3390/medicines5020046 29883402 PMC6023478

[11] Liu P, Zhao H, Luo Y. Anti-aging implications of *Astragalus membranaceus* (Huangqi): a well-known Chinese tonic. Aging and Disease. 2017; 8(6): 868–86. doi: 10.14336/AD.2017.0816 29344421 PMC5758356

[12] Auyeung KKW, Cho C-H, Ko JKS. A novel anticancer effect of Astragalus saponins: Transcriptional activation of NSAID‐activated gene. International Journal of Cancer. 2009; 125(5): 1082–91. doi: 10.1002/ijc.24397 19384947

[13] Tin MM, Cho C-H, Chan K, James AE, Ko JKS. Astragalus saponins induce growth inhibition and apoptosis in human colon cancer cells and tumor xenograft. Carcinogenesis. 2007; 28(6): 1347–55. doi: 10.1093/carcin/bgl238 17148504

[14] Wang S, Li J, Huang H, Gao W, Zhuang C, Li B, et al. Anti-hepatitis B virus activities of astragaloside IV isolated from Radix Astragali. Biological and Pharmaceutical Bulletin. 2009; 32(1): 132–35. doi: 10.1248/bpb.32.132 19122295

[15] Xu F, Cui W-Q, Wei Y, Cui J, Qiu J, Hu L-L, et al. Astragaloside IV inhibits lung cancer progression and metastasis by modulating macrophage polarization through AMPK signaling. Journal of Experimental & Clinical Cancer Research. 2018; 37(1): 207. doi: 10.1186/s13046-018-0878-0 30157903 PMC6116548

[16] Yu Y, Zhou L, Yang Y, Liu Y. Cycloastragenol: An exciting novel candidate for age‑associated diseases. Experimental and Therapeutic Medicine. 2018; 16(3): 2175–82. doi: 10.3892/etm.2018.6501 30186456 PMC6122403

[17] Ip FC, Ng YP, An HJ, Dai Y, Pang HH, Hu YQ, et al. Cycloastragenol is a potent telomerase activator in neuronal cells: implications for depression management. Neurosignals. 2014; 22(1): 52–63. doi: 10.1159/000365290 25095809

[18] Zhou R-N, Song Y-L, Ruan J-Q, Wang Y-T, Yan R. Pharmacokinetic evidence on contribution of intestinal bacterial conversion to beneficial effects of astragaloside IV, a marker compound of Astragali Radix, in traditional oral use of the herb. Drug Metabolism and Pharmacokinetics. 2012; 27: 586–97. doi: 10.2133/dmpk.dmpk-11-rg-160 22673033

[19] He Y, Hu Z, Li A, Zhu Z, Yang N, Ying Z, et al. Recent advances in biotransformation of saponins. Molecules. 2019; 24(13): 1–21. e2365. doi: 10.3390/molecules24132365 31248032 PMC6650892

[20] Liu M, Li P, Zeng X, Wu H, Su W, He J. Identification and pharmacokinetics of multiple potential bioactive constituents after oral administration of Radix Astragali on cyclophosphamide-induced immunosuppression in balb/c mice. International Journal of Molecular Sciences. 2015; 16(3): 5047–71. doi: 10.3390/ijms16035047 25751722 PMC4394464

[21] Mannelli LDC, Pacini A, Micheli L, Femia AP, Maresca M, Zanardalli M, et al. Astragali Radix: could it be an adjuvant for oxaliplatin-induced neuropathy?. Scientific Reports. 2017; 7(1): 1–13. e42021. doi: 10.1038/s41598-016-0028-x 28186109 PMC5301199

[22] Yan L-X, Guo D-A. Quantitation of astragaloside IV in rat plasma by liquid chromatography–tandem mass spectrometry. J Chromatogr B. 2005; 824(1–2): 244–48 doi: 10.1016/j.jchromb.2005.07.032 16087411

[23] Li M, Wang W, Xue J, Gu Y, Lin S. Meta-analysis of the clinical value of *Astragalus membranaceus* in diabetic nephropathy. Journal of Ethnopharmacology. 2011; 133(2): 412–419. doi: 10.1016/j.jep.2010.10.012 20951192

[24] Xiong M, He Q, Lai H, Huang W, Wang L, Yang C. Radix astragali injection enhances recovery from sudden deafness. American Journal of Otolaryngology. 2012; 33(5): 523–27. doi: 10.1016/j.amjoto.2011.12.001 22306788

[25] Rao T, Gong Y-F, Peng J-B, Wang Y-C, He K, Zhao H-H, et al. Comparative pharmacokinetic study on three formulations of Astragali Radix by an LC–MS/MS method for determination of formononetin in human plasma. Biomedical Chromatography. 2019; 33(9): 1–9. e4563. doi: 10.1002/bmc.4563 31025385

[26] Xu M, Yin J, Xie L, Zhang J, Zou C, Zou J, et al. Pharmacokinetics and tolerance of toal astragalosides after intravenous infusion of astragalosides injection in healthy Chinese volunteers. Phytomedicine. 2013; 20(12): 1105–11. doi: 10.1016/j.phymed.2013.05.004 23838148

[27] Taiwan Herbal Pharmacopeia, 2nd Edition English version. Ministry Health and Welfare, Taiwan; 2016: 39–41.

[28] Monschein M, Ardjomand-Woelkart K, Rieder J, Wolf I, Heydel B, Kunert O, et al. Accelerated sample preparation and formation of astragaloside IV in Astragali Radix. Pharm Biol. 2014; 52(4): 403–9. 10.3109/13880209.2013.839712 24171819

[29] Chu CHU E-Hu L, Lian-Wen QI, Ping LI. Transformation of astragalosides from Radix Astragali under acidic, neutral, and alkaline extraction conditions monitored by LC-ESI-TOF/MS. Chinese Journal of Natural Medicines. 2014; 12(4): 314–20. doi: 10.1016/S1875-5364(14)60062-5 24863360

[30] Knoess W, Wiesner J. The globalization of traditional medicines: Perspectives related to the European Union regulatory environment. Engineering. 2019; 5(1): 22–31. doi: 10.1016/j.eng.2018.11.012

[31] Guideline on specifications: test procedures and acceptance criteria for herbal substances, herbal preparations and herbal medicinal products/traditional herbal medicinal products. European Medicines Agency. 2011: 1–25.

[32] Li L, Zheng S, Brinckmann JA, Fu J, Zeng R, Huang L, et al. Chemical and genetic diversity of *Astragalus mongholicus* grown in different eco-climatic regions. PloS One. 2017; 12(9): 1–13. e0184791. doi: 10.1371/journal.pone.0184791 28945770 PMC5612462

[33] Dong W-W, Au D, Cao X-W, Li X-B, Yang D-J. Discriminating Astragali Radix from its adulterants using HPLC coupled with chemometric clustering techniques. Journal of Food and Drug Analysis. 2011; 19(4): 495–01.

[34] Ma XQ, Shi Q, Duan J, Dong TTX, Tsim KWK. Chemical analysis of Radix Astragali (Huangqi) in China: a comparison with its adulterants and seasonal variations. Journal of Agricultural and Food Chemistry. 2002; 50(17): 4861–66. doi: 10.1021/jf0202279 12166972

[35] Kim JH, Park S-Y, Lim HK, Park AY, Kim JS, Kang SS, et al. Quantitative evaluation of Radix Astragali through the simultaneous determination of bioactive isoflavonoids and saponins by HPLC/UV and LC-ESI-MS/MS. Bulletin of the Korean Chemical Society. 2007; 28(7): 1187–94.

[36] Kafle B, Baak J, Brede C. Quantification by LC–MS/MS of astragaloside IV and isoflavones in Astragali Radix can be more accurate by using standard addition. Phytochemical Analysis. 2020:1–8. doi: 10.1002/pca.2994 32929766

[37] Shen M-R, He Y, Shi S-M. Development of chromatographic technologies for the quality control of Traditional Chinese Medicines in the Chinese Pharmacopoeia. Journal of Pharmaceutical Analysis. 2020; Forthcoming. doi: 10.1016/j.jpha.2020.11.008 34012691 PMC8116203

[38] Zu Y, Yan M, Fu Y, Liu W, Zhang L, Gu C, et al. Determination and quantification of astragalosides in Radix Astragali and its medicinal products using LC–MS. Journal of Separation Science. 2009; 32(4): 517–25. doi: 10.1002/jssc.200800499 19160368

[39] Li W, Fitzloff JF. Determination of astragaloside IV in Radix astragali (*Astragalus membranaceus* var. *monghulicus*) using high-performance liquid chromatography with evaporative light-scattering detection. Journal of Chromatographic Science. 2001; 39(11): 459–62. doi: 10.1093/chromsci/39.11.459 11718305

[40] Zhao M, Dai Y, Li Q, Li P, Qin X-M, Chen S. A practical quality control method for saponins without UV absorption by UPLC-QDA. Frontiers in Pharmacology. 2018; 9: 1–8. e1377. doi: 10.3389/fphar.2018.00001 30618731 PMC6298191

[41] Shi J, Zheng L, Lin Z, Hou C, Liu W, Yan T, et al. Study of pharmacokinetic profiles and characteristics of active components and their metabolites in rat plasma following oral administration of the water extract of Astragali Radix using UPLC–MS/MS. Journal of Ethnopharmacology. 2015; 169: 183–94. doi: 10.1016/j.jep.2015.04.019 25917840

[42] Qiu F, Tong Z, Gao J, Wang M, Gong M. Rapid and simultaneous quantification of seven bioactive components in Radix Astragali based on pressurized liquid extraction combined with HPLC-ESI-MS/MS analysis. Analytical Methods. 2015; 7(7): 3054–62.

[43] Matuszewski BK, Constanzer ML, Chavez-Eng CM. Strategies for the assessment of matrix effect in quantitative bioanalytical methods based on HPLC− MS/MS. Analytical Chemistry. 2003; 75(13): 3019–30. doi: 10.1021/ac020361s 12964746

